# Characterization and influence of hydroxyapatite nanopowders on living cells

**DOI:** 10.3762/bjnano.9.286

**Published:** 2018-12-27

**Authors:** Przemyslaw Oberbek, Tomasz Bolek, Adrian Chlanda, Seishiro Hirano, Sylwia Kusnieruk, Julia Rogowska-Tylman, Ganna Nechyporenko, Viktor Zinchenko, Wojciech Swieszkowski, Tomasz Puzyn

**Affiliations:** 1 Central Institute for Labour Protection - National Research Institute, Department of Chemical, Biological and Aerosol Hazards, Warsaw, Poland; 2 Warsaw University of Technology, Faculty of Materials Science and Engineering, Warsaw, Poland; 3 National Centre for Nuclear Research, Material Testing Lab, Swierk, Poland; 4 National Institute for Environmental Studies, NanoTox Project, Tsukuba, Japan; 5 Polish Academy of Science, Institute of High Pressure Physics, Laboratory of Nanostructures, Warsaw, Poland; 6 A. V. Bogatsky Physical-Chemical Institute of NAS of Ukraine, Department of Chemistry of Functional Inorganic Materials, Odessa, Ukraine; 7 University of Gdansk, Faculty of Chemistry, Gdansk, Poland

**Keywords:** nanomaterials safety, biomaterials, tissue engineering, microscopic characterization, cytotoxicity, hydroxyapatite

## Abstract

Nanomaterials, such as hydroxyapatite nanoparticles show a great promise for medical applications due to their unique properties at the nanoscale. However, there are concerns about the safety of using these materials in biological environments. Despite a great number of published studies of nanoobjects and their aggregates or agglomerates, the impact of their physicochemical properties (such as particle size, surface area, purity, details of structure and degree of agglomeration) on living cells is not yet fully understood. Significant differences in these properties, resulting from different manufacturing methods, are yet another problem to be taken into consideration. The aim of this work was to investigate the correlation between the properties of nanoscale hydroxyapatite from different synthesis methods and biological activity represented by the viability of four cell lines: A549, CHO, BEAS-2B and J774.1 to assess the influence of the nanoparticles on immune, reproductive and respiratory systems.

## Introduction

Engineered nanomaterials have found applications in many sectors, including automobile, chemicals, construction, cosmetics, electronics, energy, engineering, environment, medicine, security, sports, telecommunication, textiles and transportation [1–2]. The International Organization for Standardization, in cooperation with the European Committee for Standardization, has defined nanoscale as a size range from approximately 1 to 100 nm. A nanomaterial is defined as a material (natural, incidental or manufactured) containing particles, in an unbound state, or as an aggregate (object composed of strongly or fused bounded particles), or as an agglomerate (composite of weakly or medium strongly bound particles), in which for 50% or more of the particles in the number size distribution one or more external dimensions is at the nanoscale [3]. According to the current state of knowledge, aggregates and agglomerates of the nanoobjects (NOAA) that are bigger than 100 nm can exhibit properties (including toxicological) different from those of non-nanoscale (bulk) materials [4].

Interest in the use of nanomaterials for biomedical applications is constantly growing due to their unique physical, chemical, biological and mechanical properties [5–6]. However, interactions between nanoparticles (NPs) and the biological environment are not yet fully understood. Structures such as human skin or lungs are in constant contact with the environment and are thus exposed to nanoobjects. Lack of knowledge about nanoparticle effects on cell viability is a significant barrier in the application of novel NPs in medicine and biology. Adverse effect of NOAA on living organisms may be influenced by many factors such as size, shape, solubility, exposure time, surface charge, surface area, crystallinity, agglomeration or chemical composition [7–9]. Since the properties of nanoparticles are size-dependent, it might be prudent to assume the same about their biotoxicity. Because of their size, nanoobjects are able to be internalized by living cells and affect basic cellular processes such as metabolism, proliferation, differentiation or lysis [10].

Hydroxyapatite (Hap, Ca_10_(PO_4_)_6_(OH)_2_) is a calcium phosphate, structurally and chemically similar to the mineral phase of human bone and teeth. Due to its high biocompatibility and bioactivity, it has been successfully applied in the manufacturing of cosmetics and hygiene products, as well as in bone-tissue engineering and regenerative medicine. The use of nanosized hydroxyapatites in biomedical applications is constantly growing due to their good mechanical properties and enhanced efficiency of gene transfection in drug delivery. Calcium phosphates are sensitive to the preparation conditions [11–15]. They can be manufactured using many different methods, such as hydrothermal synthesis, sol–gel synthesis, wet-chemical precipitation and microwave processing [16–19]. Many studies have shown that HAp can cause different biological responses depending on its physicochemical properties [20–22]. It is also important to mention that the source of nanoparticle exposure could be not only the final product (from degradation or wear) but nanoparticles can also be released during the manufacturing process.

Several studies have been previously conducted to assess the toxicity of nanoscale hydroxyapatite, but the number of samples, biological models and characterization methods provided encompassed much less than the scope of this work. Previous studies have focused mainly on the impact of shape, size or surface charge of the nanoparticles alone [20,23–25]. An approach limited to one or two features significantly limits the insight of underlying mechanisms that affect living cells.

There is a wide range of available cell lines to study possible organism reactions and cytotoxicity mechanisms, such as endothelial, neural, hepatic, phagocytic or cancer cells [26–27]. Still, systematic studies describing different properties of nanopowders and their effect on different cell lines are missing. The aim of this study was to perform a complex, multi-technique physicochemical characterization of ten types of hydroxyapatite nanopowders and to determine their property-dependent influence on living cells. This allowed for the recognition of the impact of the nanoparticles on cell models mimicking immune, reproductive and respiratory systems of living organisms.

## Experimental

### Materials

Ten hydroxyapatite powders were bought or manufactured exclusively for this study. The powder-selection criteria were different manufacturing methods that resulted in a diversity of the powder parameters. Six HAp powders used in this study were produced in the Laboratory of Nanostructures (Institute of High Pressure Physics of the Polish Academy of Sciences, Warsaw, Poland), two were particularly manufactured for this study by Bogatsky Physical-Chemical Institute of NAS of Ukraine (Department of Chemistry of Functional Inorganic Materials, Odessa, Ukraine), two were prepared by using a wet-chemical laboratory method, and two kinds of hydroxyapatite powder were purchased from Sigma-Aldrich (see Table 1). The HAp samples from Sigma-Aldrich were manufactured using a combustion chemical vapour condensation process (a method developed by the company), trade-named NanoSpray Combustion™ (abbreviated HApSA in this article) and with 5% silicone admixture (HApSA+Si). Laboratory-made HAp samples were produced through a wet-chemical method without any heat treatment, and additionally obtained in the form of a spray-dried (rapid drying with a hot gas) aggregated micrometer-size powder. Both samples differed in agglomerate size (F201 and F202). HAp samples prepared by the Laboratory of Nanostructures were produced through microwave solvothermal synthesis [28–29] at a pressure of 3 bar and with different reaction times. The first solution was treated with high-energy microwave radiation for 90 s (GoHAP90s), the second solution was treated for 300 s (GoHAP300s), and the third solution was treated for 600 s (GoHAP600s). The fourth solution was also treated for 90 s, with an additional heat treatment at 375 °C. All GoHAP samples were freeze-dried after synthesis. Samples of nanoscale hydroxyapatite and fluorohydroxyapatite from the Bogatsky Institute were synthetized at a temperature of 300 °C with the NaNO_3_/KNO_3_ eutectic as reaction medium [14], with and without the admixture of fluorine (CaHAP300 and CaHFAP300, respectively) and with subsequent filter drying. Hydroxyapatites from the Laboratory of Nanostructures and from the Bogatsky Institute were synthesized with a calcium deficiency. The Ca/P atomic ratio of the obtained HAp samples is very similar to the ratio present in tooth enamel (1.62) [30].

**Table 1:** Studied calcium phosphates.

sample	approximate average particle size [nm] provided by the producer	Ca/P atomic ratio provided by the producer

HApSA	100	1.65
HApSA+Si	100	1.65

F201	20 nm (in 2.5 ± 0.5 μm agglomerates)	1.66
F202	20 nm (in 5 ± 1 μm agglomerates)	1.67

GoHAP90s	9	1.59
GoHAP375C	16	1.59
GoHAP300s	21	1.61
GoHAP600s	32	1.61

CaHAP300	50	1.63
CaHFAP300	80	1.59

### Characterization methods

Based on the work of Oberdörster et al. [7] and OECD recommendations [31], various features including density, morphology, average particle size, particle shape expressed by aspect ratio, state of agglomeration, specific surface area, crystallinity, phase purity, stoichiometry, zeta potential and pH value were chosen for correlating the parameters with biological activity.

#### Density measurements

Density (ρ) measurements were performed using a helium pycnometer (AccuPyc II, model 1340; Micromeritics, Australia) using an in-house procedure [32].

#### Specific surface area

The specific surface area (SSA) of the samples was determined through the Brunauer–Emmett–Teller (BET) method (AccuPyc, model Gemini 2360; Micromeritics Gosford, Australia).

#### Transmission electron microscopy

Transmission electron microscopy (TEM, JEOL JEM-2010, JEOL Ltd, Tokyo, Japan, JED 2300 series analyzer) was used to determine average size (AS) and the shape of the particles [33]. Hydroxyapatites were observed at an accelerating voltage of 100 kV with a LaB_6_ cathode. Samples were prepared by suspending a small quantity of nanopowder in ethanol, followed by 5 min of ultrasonic treatment. The samples were then left for about 10 min to evaporate the ethanol. Afterwards, they were dripped on the surface of carbon-coated copper grids. Sizes of crystallites were measured with the ImageJ software [34], using representative TEM images. Average values were calculated from no less than 200 particles. Pixel size was 1.8 nm × 1.8 nm.

#### Scanning electron microscopy

Morphology and agglomeration state of the HAp samples were examined with a HITACHI S5500 for nonconductive materials (accelerating voltage = 3–5 kV). The samples were prepared by droplet evaporation. A small quantity of powder (1–5 μg/mL) was suspended in ethanol and remained on the SEM stage until the complete evaporation of ethanol. The maximum Feret diameter (MF) of HAp NOAA was calculated from at least 1500 objects from 400× magnified images. Analyses were performed automatically from representative SEM images using the ImageJ software [34]. SEM in combination with an X-ray microanalyser (EDS, energy dispersion spectroscopy) was used to carry out chemical pre-analysis. EDS was carried out in dark-field (accelerating voltage = 8 kV, 2 min scan, ca. 10,000 counts) and quantitative analysis was done taking into account at least 15 different agglomerates for each sample.

#### Atomic force microscopy

Atomic force microscope (AFM) was used for topography imaging, surface evaluation at the nanoscale and evaluation of the particle shapes [35]. The sample preparation protocol was as follows: A water suspension of nanopowder was prepared at a concentration of 0.1 mg/mL. A droplet of the suspension was dripped onto the surface of a freshly cleaved mica disc (Ted Pella) and left in a vacuum dryer (VO 200, Memmert) for 1 h prior to imaging.

**Figure 1:**
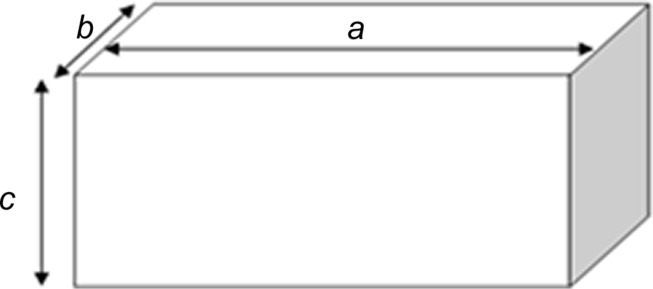
Aspect ratio for a three-dimensional object: 
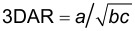
.

Topography measurements were made in air. 1 µm × 1 µm scans were taken with 0.8 Hz scan rate using a silicon probe (*k* = 40 N/m, *r* = 10 nm) from Bruker AFM Probes [36]. Image analysis and measurement of length, width and height of the HAp particles was performed with the Gwyddion software [37]. The tip-broadening error was removed by using a method described by Kacher and co-workers [38]. Based on the obtained dimensions, the aspect ratio in three dimensions (3D AR, see Figure 1) was calculated and used for particle-shape evaluation. For a spherical shape, the value of 3D AR approaches 1. A higher value represents a more elongated shape.

#### Dynamic light scattering

The average size of HAp nanoobjects in water (*L*), pH value and zeta potential (ζ) of particles were determined by using dynamic light scattering (DLS, Malvern Instruments Zetasizer Ltd, Spectris). Samples were prepared in 0.02% water solutions with addition of 0.1% Pluronic (used in cell assays, Sigma-Aldrich Co. LLC) at *T* = 25 °C and the analysis was performed 1 h after suspending the HAps.

#### X-ray diffraction

The phase composition of the samples was examined by using X-ray diffraction (Rigaku Ultima, Cu Kα_1_ radiation, λ = 1.54059 Å, 26 mA and 40 kV). The patterns were collected at standard temperature in the 2θ range of 10–60° and with a step size of 0.02° at room temperature. Obtained spectra were imported into „Match!” software and compared to the reference spectra from the ICDD PDF-2 database (The International Center for Diffraction Data, 2015) for calcium phosphate hydroxide (hydroxyapatite, syn, base number 00-009-0432 [PDF 9-432]). The analysis of diffractograms was supported by the TOPAS R software (Bruker-AXS) [39]. Phase purity (*X*_p_) of the samples was presented as the percentage content of reference HAp in relation to the total crystal phase content of the sample [40–41] containing both identified foreign phases and unidentified reflexes in the range of 10 to 60° (2θ). Crystallinity (*X*_c_) refers to the degree of structural order in a solid. It is a concept that integrates diffraction domain size, crystal strain and crystal defects. The crystallinity percentage of the samples was measured by using the following equation [42–44]:

[1] $$X_{\text{c}}\left(\%\right)=\frac{\sum A_{\text{c}}}{\sum A_{\text{c}}+\sum A_{\text{A}}}\cdot 100,$$

where Σ*A*c is the sum of the area under all of the reflections in the diffraction pattern, and Σ*A*_c_ + Σ*A*_A_ is the summarized area under all crystalline and amorphous reflections of HAp.

#### Cell assays

Four different cell lines were used to assess nanoparticle cytotoxicity: chinese hamster ovary cell line (CHO) showing the effect on cells of the reproductive system, mouse monocyte macrophage cell line (J774.1) showing the effect on cells of the immune system, human bronchial epithelial cell line (BEAS-2B) and human lung adenocarcinoma epithelial cell line (A549), both showing the effect on cells of the respiratory system, which is important for inhalation-exposure assessment during preparation and processing of nanoscale hydroxyapatites.

#### Cell culture

All cells were maintained in 35 or 60 mm Φ culture dishes and passaged when 85–90% confluence was attained, i.e., every 2–5 days. CHO cells were cultured in F-12 nutrient mixture (Life Technologies Corporation). BEAS-2B and A549 cells were cultured in DMEM (Dulbecco's MEM, Wako Pure Chemical Industries, Ltd). J744.1 cells were cultured in RPMI 1640 medium with 2 mM of glutamine (Life Technologies Corporation). All culture media were supplemented with 10% heat-inactivated fetal bovine serum (FBS, HyClone™), 100 μg/mL streptomycin and 100 μg/mL penicillin. The cells were cultured at 37 °C in an incubator with a humidified atmosphere containing 5% CO_2_. For the passage procedure, 0.05% Trypsin–EDTA with phenol red was used (Life Technologies Corporation). HAp powder suspensions were ultrasonicated before being brought into the cell environment.

#### WST-8 assay

The viability of cells in the presence of hydroxyapatite in different concentrations was analysed using a colourimetric assay for quantification of the cleavage of tetrazolium salt WST-8 (Dojindo Molecular Technologies, Inc, Japan) by mitochondrial dehydrogenases. The WST-8 assay is a development of the MTT assay, which is used for determining the cells metabolic activity. It is a colourimetric assay based on the extracellular reduction of tetrazolium salt WST-8 produced by mitochondrial NADH or NADPH to the water-soluble, strongly coloured formazan. The concentrations under study were 10, 25, 50, 75, 100, 125, 150, 200, 250 and 300 µg/mL, but for better clarity of presented results only 10, 100, 200 and 300 μg/mL are shown in this paper. CHO, BEAS-2B, A549, and J774.1 cells were separately cultured with various concentrations of hydroxyapatite and 0.1% of Pluronic (used as a stabilizer of cell membranes protecting from membrane shearing and additionally acts as an anti-foaming agent; Pluronic F68 - Sigma-Aldrich Co. LLC) in a 24-well culture dish for 24 h, then washed with PBS and moved to 96-well dish, after which cell viability was evaluated. The numbers of living cells measured are presented as a percentage relative to the negative control (100%), as determined using the WST-8 assay. Results above 100% are taken to show a stimulated growth, and results below 100% to show a growth inhibition. Cell viabilities equal or less than 50% were assumed to indicate a toxic effect [45]. All quantitative WST-8 tests were carried out in triplicate. The data were expressed as means ± standard deviation.

#### Confocal laser scanning microscopy

A confocal laser scanning microscope (CLSM, Leica Confocal Microscope, SP5 Microscope model: DMI6000, Laser: Argon 10%, line length 511–520 nm) equipped with cell life support unit was used to observe interactions between cells and particles. Cells were incubated overnight in glass-bottom Petri dishes. Single plane images were collected after 4 h of cell exposure to the particles.

#### Statistical analysis

Statistical significance between samples and control cells was analysed by one-way ANOVA and Dunnett’s post-hoc test (*P < 0.05, **P < 0.01, ***P < 0.001) [46]. Analysis was supported by KyPlot software [47]. To evaluate the manufacturing methods and to analyse the interaction mechanisms of HAp with living cells, simple statistical correlations and regressions of physicochemical properties with biological activity expressed by cell viability were made (significance [p] set at α = 0.05).

## Results and Discussion

The physical properties of the investigated HAp samples are summarized in Table 1 and Table 2. The stoichiometric ratio Ca/P of most samples was 1.67 or close to this ideal value of hydroxyapatite (see Ca/P in Table 1). Positive and negative deviation from this number suggests a lower crystallinity of the samples, which was proven by XRD analysis (see *X*_c_ in Table 2).

**Table 2:** Physicochemical parameters of the studied HAp samples.

sample	BET		TEM	SEM	AFM	shape of particles^c^	DLS			XRD	
	ρ [kg/m^3^]	SSA [m^2^/g]	AS [nm]^a^	MF [µm]^b^	3D AR		ζ potential [mV]	*L* [µm]	pH	*X*_p_	*X*_c_

HApSA	3.17 ± 0.02	18.58	50.88 ± 24.83	2.33 ± 3.93	1.36	spherical	−1.02	5.60	7.18	93.1%	95.1%
HApSA+Si	2.96 ± 0.09	15.51	30.48 ± 9.67	1.44 ± 2.54	1.32	spherical	0.26	4.34	7.69	28.6%	64.2%
F201	2.93 ± 0.01	112.05	23.19 ± 9.61	2.21 ± 1.34	5.84	needle-like	−2.11	3.60	7.06	78.7%	97.3%
F202	2.95 ± 0.02	98.99	21.32 ± 9.78	3.32 ± 1.97	2.18	ellipsoidal	−2.95	5.55	6.93	78.5%	99.1%
GoHAP90s	2.91 ± 0.01	163.92	13.79 ± 6.43	2.86 ± 6.26	2.41	elongated ellipsoidal	0.22	7.88	7.19	83.8%	86.3%
GoHAP375C	2.99 ± 0.01	108.44	29.53 ± 8.16	3.63 ± 7.52	1.68	ellipsoidal	−1.1	4.08	7.16	88.5%	91.6%
GoHAP300s	3.03 ± 0.01	66.28	33.29 ± 14.28	5.13 ± 8.20	1.26	spherical	−1.86	7.18	6.98	88.4%	87.4%
GoHAP600s	2.99 ± 0.01	102.68	32.06 ± 10.07	2.37 ± 5.34	1.13	spherical	−3.93	5.55	6.78	83.9%	89.6%
CaHAP300	2.64 ± 0.05	30.77	50.79 ± 34.75	1.94 ± 2.65	2.30	elongated ellipsoidal	−16.21	0.80	7.52	26.5%	78.1%
CaHFAP300	2.65 ± 0.03	19.95	60.06 ± 32.58	1.86 ± 2.67	3.08	flake-like	−17.02	0.69	7.53	14.2%	70.5%

^a^mean ± standard deviation for *n* = 200; ^b^mean ± standard deviation for *n* > 1500; ^c^based on 3D aspect ratio from analyses of AFM and TEM images.

The only exception is HApSA+Si. Here, the manufacturer has provided a ratio of pure HAp, not of its mixture with silicone, which could be incorporated into the crystal structure. GoHAP375C, despite its low stoichiometry, had a high crystallinity, which may be the result of thermal processing and the ordering of the crystals. The nanoparticles had a lower density than their microscale counterparts. Literature data show that the density of nanoscale hydroxyapatite is about 3.05 g/cm^3^, while the density of microscale hydroxyapatite powder is about 3.16 g/cm^3^ [48]. HApSA had the highest density of 3.17 g/cm^3^, close to the value of microscale HAp. Along with HApSA+Si, CaHFAP300 and CaHAP300 had a notably small surface area (in order of increasing SSA value: HApSA+Si < HApSA < CaHFAP300 < CaHAP300). The lowest density among the tested materials was calculated for CaHAP300 and CaHFAP300 powders (ca. 2.64 g/cm^3^ and 2.65 g/cm^3^, respectively) which in combination with their small surface areas may indicate a lack of phase purity. The density of the other samples ranged from 2.93 to 3.03 g/cm^3^, and all of them had relatively high surface areas ranging from 66.3 m^2^/g to 163.9 m^2^/g, indicating a small size of the particles confirmed by bright-field TEM imaging (see AS values in Table 2). Since the particle sizes in the range of 1–10 nm are comparable to the size of DNA, they may cause toxic and mutagenic effects [49–50]. Therefore, it was calculated how many crystals in this size rang are on the analysed TEM images. Only F201 (8%), F202 (15%) and GoHAP90s (40%) exhibited a content of such small crystallites above 1%. The average particle size determined with the use of TEM imaging did not differ significantly from the values given by the manufacturers.

### Microscopy observations

**Figure 2:**
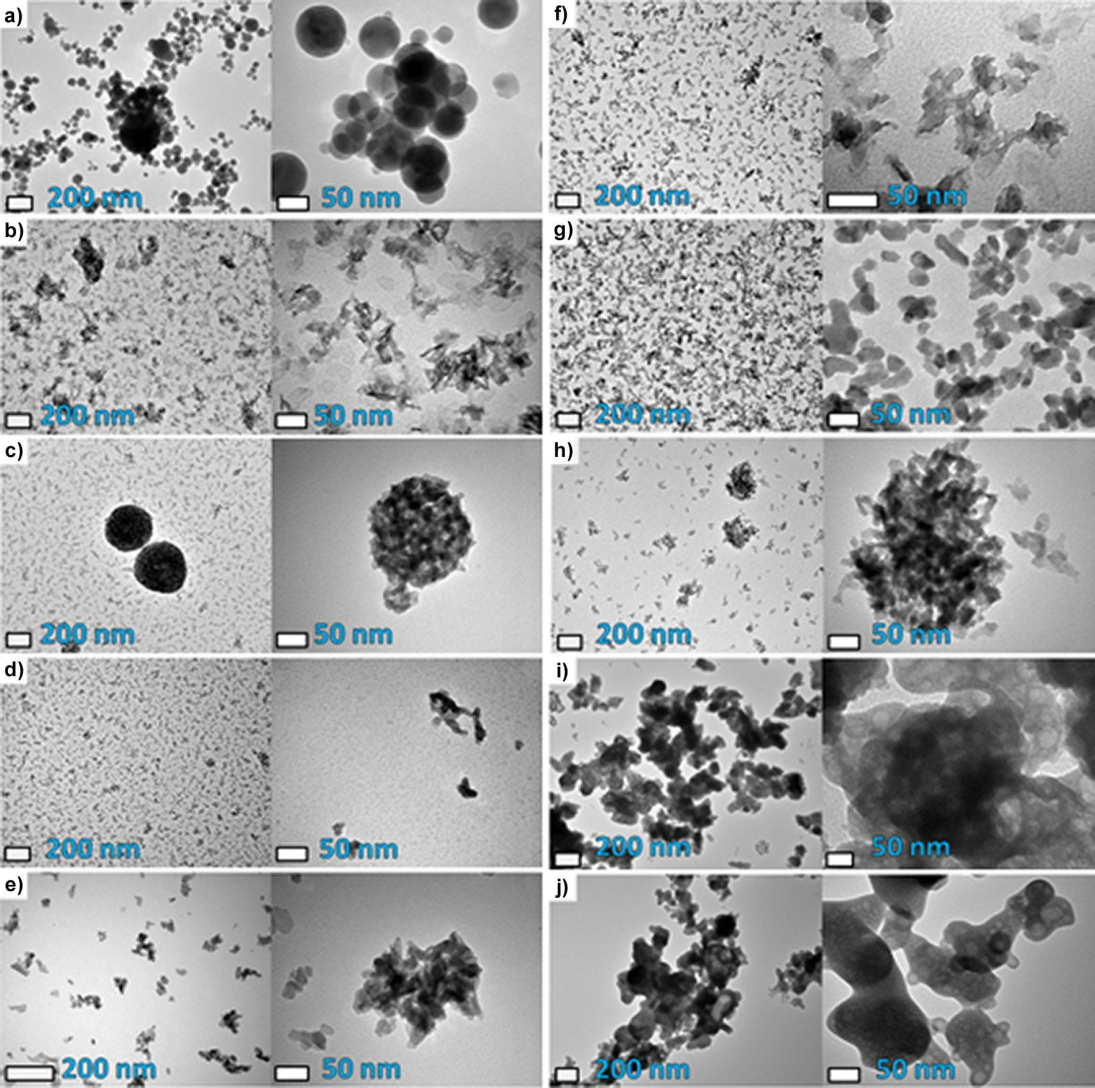
TEM micrographs of a) HApSA, b) HApSA+Si, c) F201 d) F202, e) GoHAP90s, f) GoHAP375C, g) GoHAP300s, h) GoHAP600s, i) CaHAP300, j) CaHFAP300. Scale bars on the images from the left to right show 200 nm and 50 nm.

TEM micrographs (Figure 2) combined with the 3D aspect ratio calculated from AFM measurements allowed us to evaluate the shape of the particles. In general, the smaller a particle was, the more elongated was its shape. CaHAP300 and CaHFAP300 were the only exceptions. With relatively large particles, their 3D AR suggest extended ellipsoidal and flake-like shapes, but it must be noted that the shape factor is only a simplified way to describe a three-dimensional object using one value and can also include more complex shapes such as flakes, stars, irregular granules, sponges and polyhedrons.

**Figure 3:**
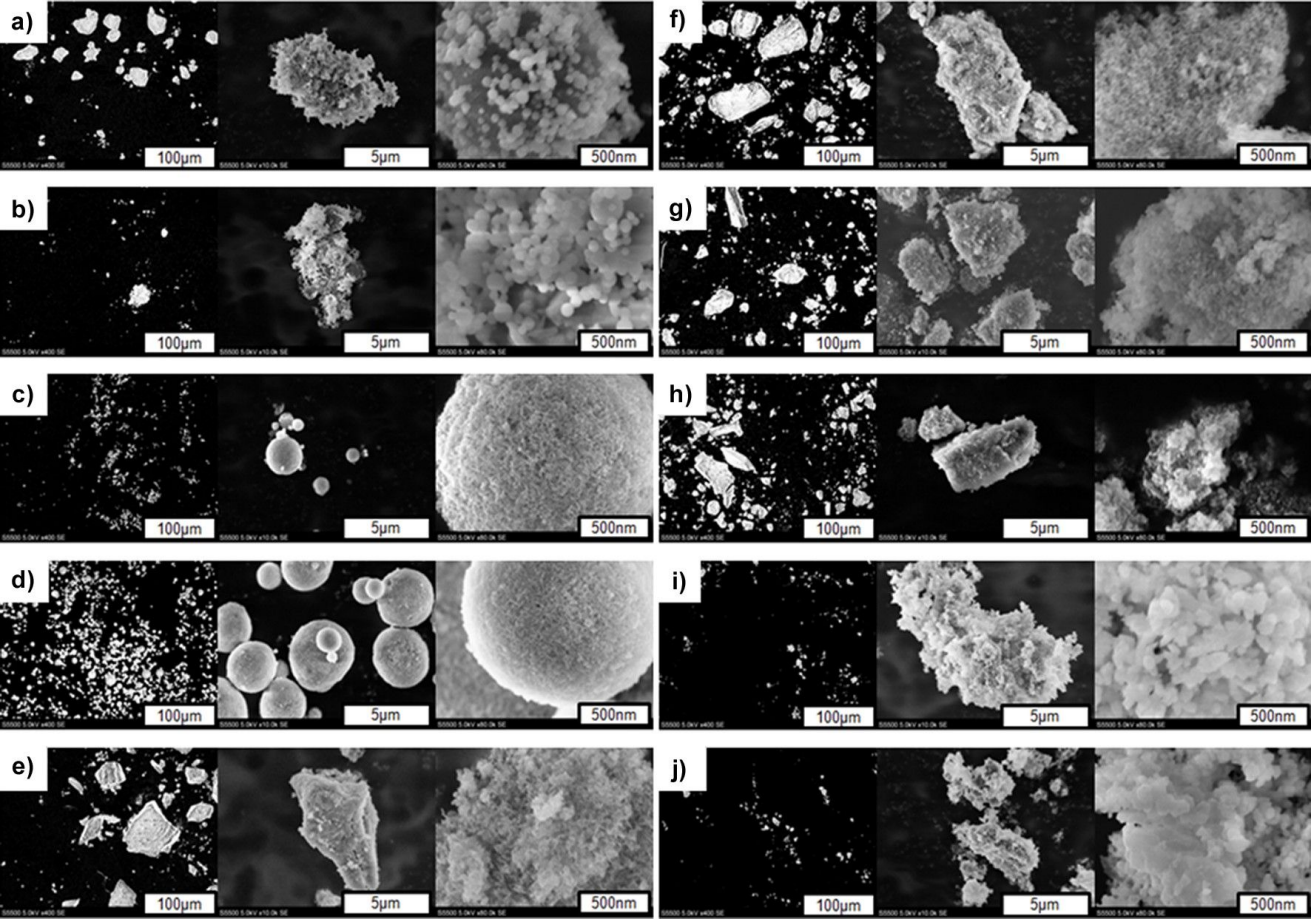
SEM micrographs of a) HApSA, b) HApSA+Si, c) F201 d) F202, e) GoHAP90s, f) GoHAP375C, g) GoHAP300s, h) GoHAP600s, i) CaHAP300 and j) CaHFAP300. Scale bars on the images from the left to right show 100 µm, 5 µm and 0.5 µm.

SEM micrographs (Figure 3) of the investigated samples allowed for the determination of the state of agglomeration. The HAp samples were composed of irregular agglomerates, the only exceptions being the F201 and F202 samples (Figure 3c,d). Due to spray drying they formed stable, spherical agglomerates with a narrow size distribution. All GoHAp samples exhibited aggregates with sharp edges and agglomerates with a very wide size distribution (visible in the results as high values of standard deviation). HApSA and HApSA+Si form irregular agglomerates built from bigger particles covered with smaller ones.

It can be clearly stated that all the tested nanoscale hydroxyapatites underwent agglomeration, regardless of the manufacturing technique of the powders. It is worth to note that, as stated earlier, spray drying yielded powders with the narrowest size distribution of all tested HAps (Table 2).

**Figure 4:**
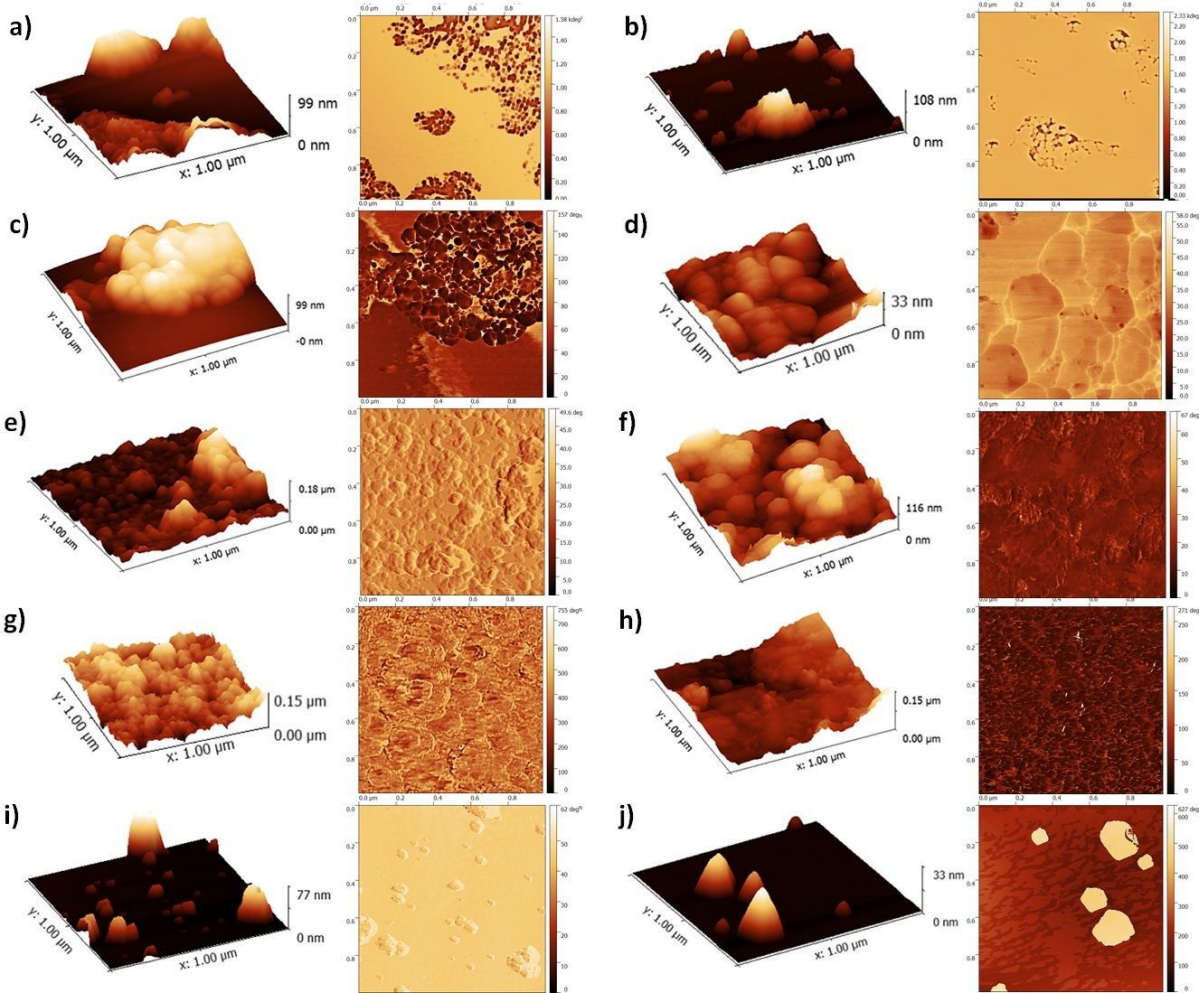
AFM images of 3D and 2D topography: a) HApSA, b) HApSA+Si, c) F201 d) F202, e) GoHAP90s, f) GoHAP375C, g) GoHAP300s, h) GoHAP600s, i) CaHAP300 and j) CaHFAP300. Scan size 1 µm × 1 µm.

Obtained SEM data were further verified using atomic force microscopy. It is worth to note that AFM has proven to be a powerful visualization technique for a wide range of nanomaterials and nanoscale changes in the materials [51–53], including qualitative and quantitative analysis of nanopowders [54]. AFM allowed for 3D topography reconstruction and phase-contrast acquisition yielding for deeper insight into nanoscale features of the tested materials. It is important to note that nanoparticle imaging requires scanning probes with a small tip radius, otherwise the acquired data might be inaccurate. For this purpose, we used a scanning probe with a radius below 10 nm. AFM topographical maps (Figure 4) were in good conformity with SEM images. All HAp powders underwent agglomeration independently from their size and shape. Phase-contrast maps exposed particle boundaries more clearly than the topographical images. Hence, these maps were used for shape quantification of the HAp NPs (Table 2).

AFM imaging of nanopowders is a challenging task. The phase-contrast maps acquisition parameters were changed accordingly to record high-resolution, quality images. The difference in lateral size of the tested powders and their different binding forces to the mica disc constituted additional factors to be considered during image acquisition. Images presenting a higher level of details at different size scales were included to avoid the risk of data loss linked to the setting of an arbitrary phase-contrast base value for one sample image size.

### Physicochemical evaluation of nanoparticles

**Figure 5:**
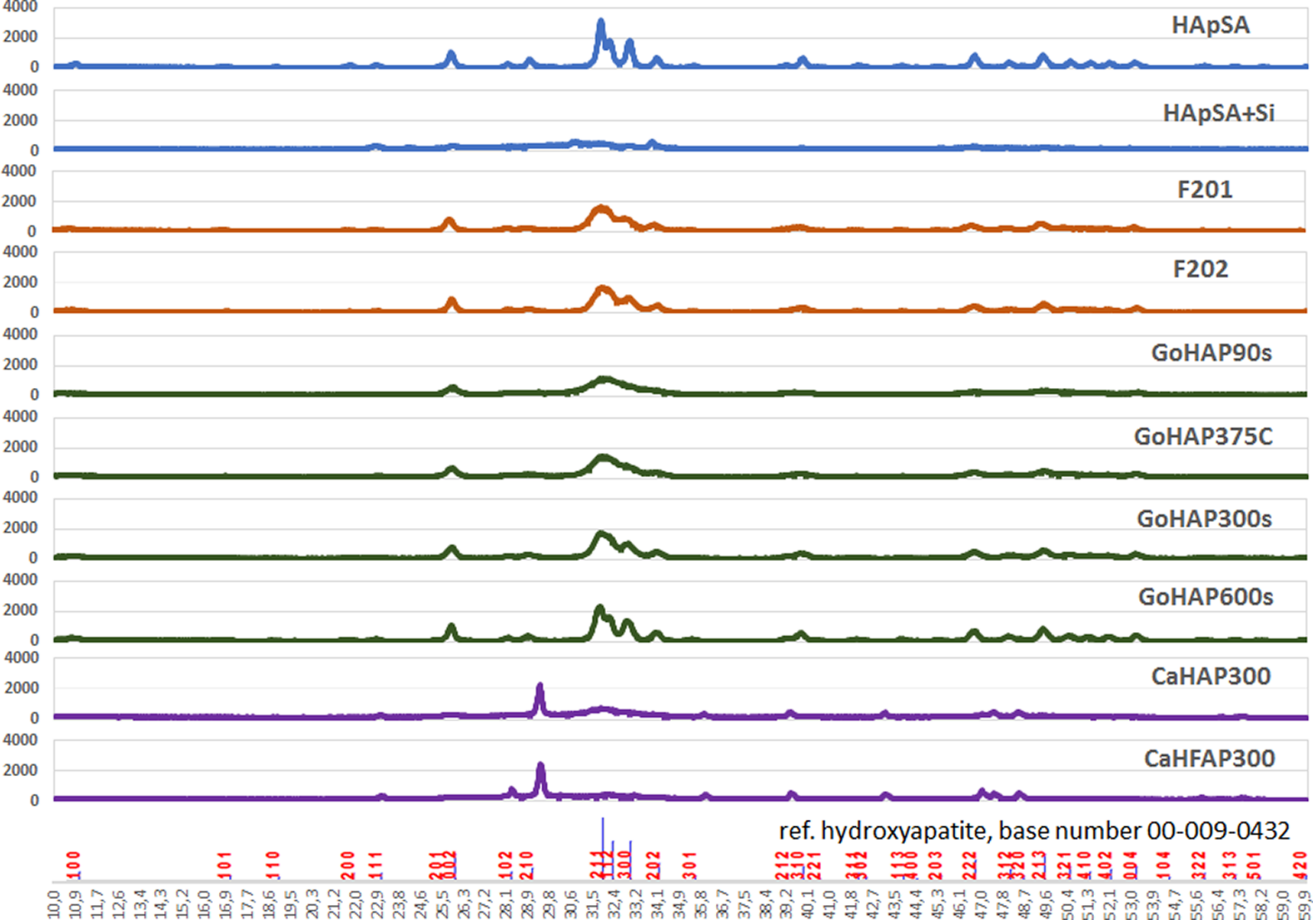
From the top, powder XRD patterns of: a) HApSA, b) HApSA+Si, c) F201 d) F202, e) GoHAP90s, f) GoHAP375C, g) GoHAP300s, h) GoHAP600s, i) CaHAP300, j) CaHFAP300 and at the bottom - reference HAp from ICDD PDF-2 database (entry number 00-009-0432) with Miller indices (h k l). 2Θ range: 10-60.

Interactions between particles in suspension depend strongly on their zeta potential. Particles having zeta potential values below ±10 mV are considered neutral, with a strong tendency to agglomerate [55]. The zeta potential values of most examined samples (Table 2) were very small, ranging from 0.22 mV to −3.93 mV, which resulted in average sizes of the nanoobjects in water ranging from 3.6 to 7.9 µm. Higher values were exhibited only by the samples CaHAP300 and CaHFAP300, with −16.21 mV and −17.02 mV respectively, which resulted in smaller nanoobject sizes of 0.8 and 0.7 µm. Sizes measured with DLS in water were slightly higher than the SEM-based values measured in vacuum, which is understandable, because in DLS large agglomerates mask the presence of small particles and aggregates, giving higher average values. Once again, the CaHAP300 and CaHFAP300 samples showed a marked difference from the rest of the powders, with their nanoobjects having relatively large sizes in vacuum (by SEM imaging: 1.94 and 1.86 µm respectively), which, compared to DLS results, suggest their higher solubility in water. The pH values of GoHAP600s, F202, and GoHAP300s in water were slightly lower than neutral (6.78, 6.93, 6.98, respectively). For F201, GoHAP375C, HApSA, and GoHAP90s the values were slightly higher than neutral (7.06, 7.16, 7.18, 7.19, respectively). CaHAP300, CaHFAP300, and HApSA+Si exhibited higher pH values (7.52, 7.53, 7.69, respectively). During the EDS investigations, sodium ions were detected in the CaHAP300 and CaHFAP300 samples (1.15 and 1.12 atom %, respectively), which was probably a residue from the molten-salt synthesis [56–57]. The presence of fluoride has been confirmed in the CaHFAP300 sample (2.13 atom %) and the presence of silicon in HApSA+Si (2.31 atom %). Silicon was also found in CaHFAP300 (0.05 atom %), which could have been an impurity from glass used during the product synthesis. The presence of carbon and lighter elements could not be determined by EDS. A more detailed analysis was conducted with XRD (Figure 5), which was used for the determination of additional non-HAp crystalline phases, of phase purity (*X*_p_) and of the degree of crystallinity (*X*_c_). The collected diffraction patterns were compared to the reference data from the ICDD PDF-2 database [00-009-0432] (Table 2). An additional tricalcium silicate phase (ICDD 00-070-1846, Ca_3_SiO_5_; 9.13%) [58] was found in HApSA+Si. Both CaHAP300 and CaHFAP300 had a high content of calcium carbonate (ICDD 00-072-1937, CaCO_3_: 41.22% and 47.84%, respectively) [59]. CaHFAP300 also had a fluorite phase (ICDD 00-075-0363, CaF_2_ 11.36%) [60]. The carbonate content may be associated with unreacted calcium carbonate derived from incomplete product synthesis. Deviations from the expected chemical compositions of the mentioned samples explained the different zeta potential values (due to the content of the carbonate groups), lower density and higher solubility of CaHAP300 and CaHFAP300. Chemical compounds with additional phases exhibited a low degree of crystallinity and phase purity. Their diffraction patterns were visibly different from the others. The HApSA+Si diffraction pattern shows a low degree of crystallinity.

The sample most compatible with the reference hydroxyapatite is HApSA, which has the smallest surface area, relatively large particles and the highest density of all samples. Samples F201, F202, GoHAP90s, GoHAP375C, GoHAP300s and GoHAP600s have a lower phase purity, but this may be due to small particle sizes or minor impurities (detection limit for the method is 5%).

### Cell viability

**Figure 6:**
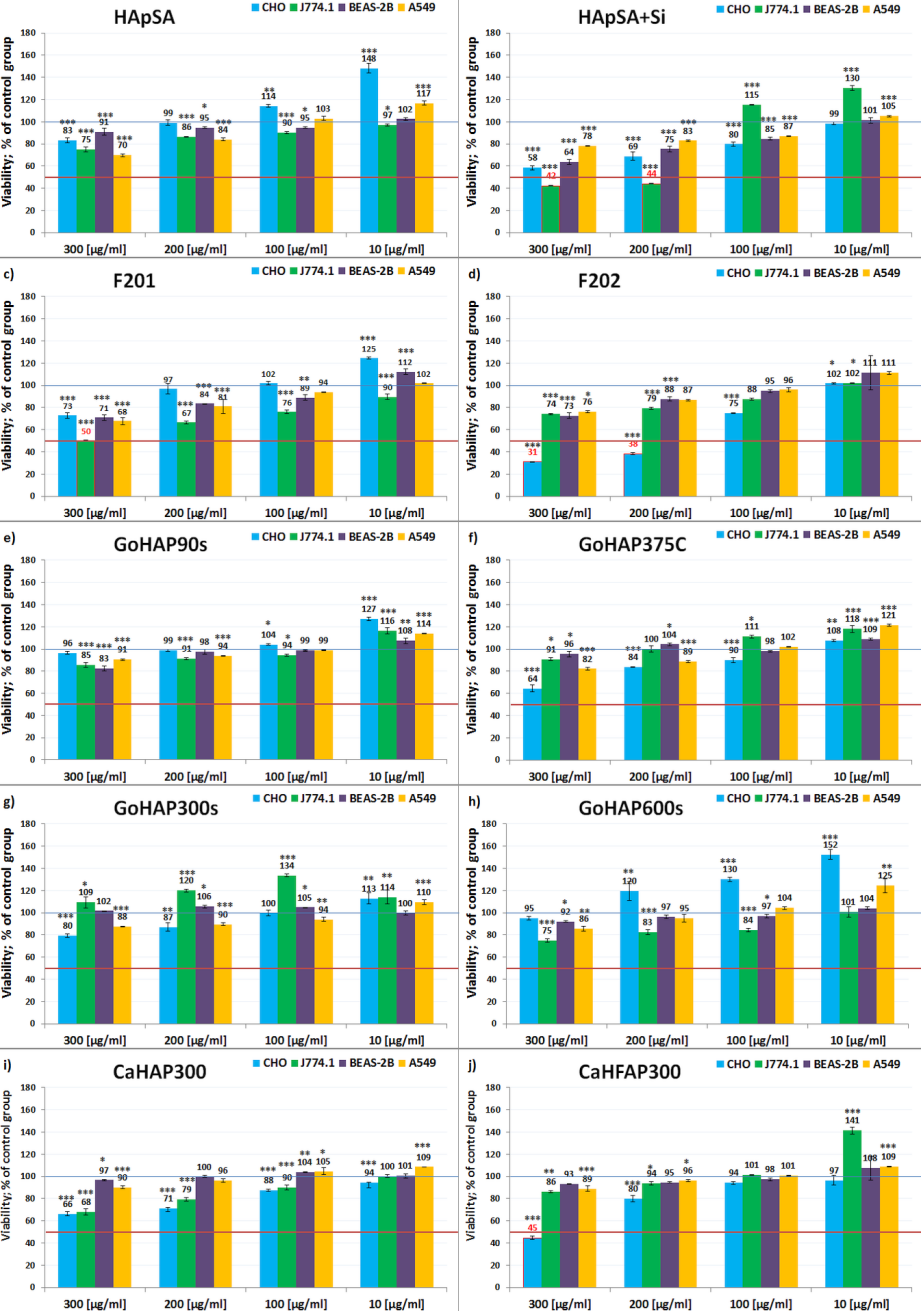
Viability of CHO, BEAS-2B, A549 and J774.1 cells, expressed as a percentage of negative control, after 24 h of exposure to 300, 200, 100 or 10 μg/mL nanoobjects of a) HApSA, b) HApSA+Si, c) F201 d) F202, e) GoHAP90s, f) GoHAP375C, g) GoHAP300s, h) GoHAP600s, i) CaHAP300, j) CaHFAP300. In every figure, a blue line shows the level of control (100% of the living cells after 24 h) and the red line shows the toxicity threshold (50% of the living cells after 24 h).

WST-8 colorimetric assays (Figure 6) were conducted to measure the cell viability in the presence of different concentrations of hydroxyapatite nanoobjects (10–300 µg/mL). The number of cells in the pool exposed to the hydroxyapatite after 24 h of incubation was compared to the number of cells in the control pool, not exposed to HAp (negative control).

Four of tested powders should be recognized as toxic, namely F201, F202, HApSA+Si, and CaHFAP300. Similarly, the CaHAP300 sample has also shown relatively low cell viability results, but did not exceed the established toxicity threshold within the test concentration range. HApSA exhibits a toxic effect in the tested range only after having been doped with silicon. Only small differences between the GoHAP hydroxyapatites can be seen; the particle size associated with the controlled reaction time and the annealing of the material at 375 °C slightly negatively influenced the interaction with cells, but no toxic effect was observed. Most of the materials induced cell growth at low concentration levels. The overdose effect is best seen at 300 µg/mL.

For A549 cells, no toxicity was noted in the tested concentration range and there was no strong cell-growth inhibition. BEAS-2B cells were the least affected by the presence of HAp nanoparticles, and no strong growth inhibitions were noted in this case as well. A toxic effect on the proliferation of macrophage cells J774.1 was noticed above 200 µg/mL for HApSA+Si and at 300 µg/mL for HApSA+Si and F201. In the case of CHO cells, HAp F202 had a toxic effect at concentrations of 200 and 300 µg/mL. The presence of CaHFAP300 also caused a toxic effect at a concentration of 300 µg/mL.

### Particle uptake and HAp–cell interactions

**Figure 7:**
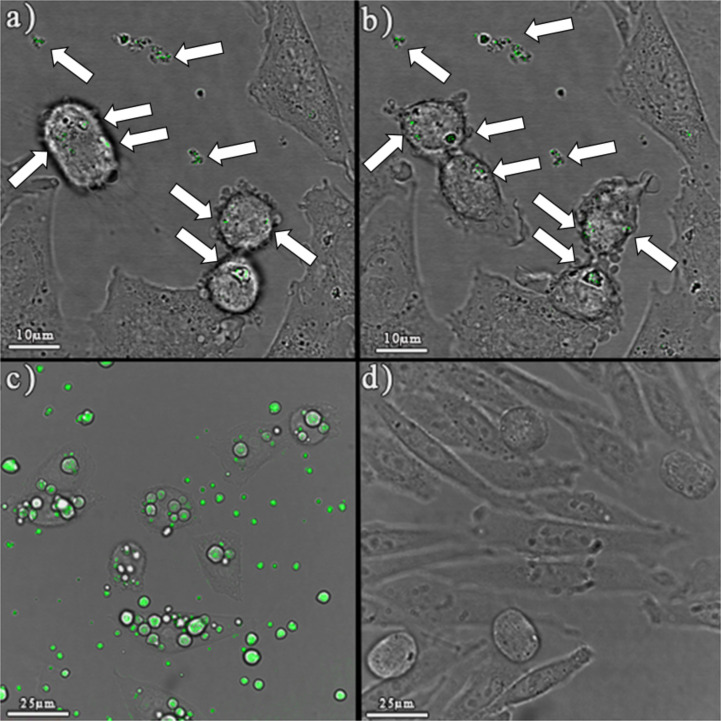
CLSM image of CHO cells and visible GoHAP90s (50 µg/mL) aggregates and agglomerates. A cell is visible a) during ) and b) after mitotic division on the left side of both images (scale bar = 10 μm). White arrows point at HAp nanoobjects. c) CLSM image of CHO cells and visible aggregates and agglomerates of F202 (100 µg/mL) hydroxyapatite, and d) image presenting the control CHO cells without any HAp additions. No staining was used. Hydroxyapatite nanoparticle agglomerates are clearly visible (scale bar = 25 μm).

CHO cells were chosen for the visualization of cell interaction with HAp, since these cells displayed the greatest susceptibility in the viability tests. HAp agglomerates were clearly visible because of their ability to reflect a laser beam in the used wavelength range. Figure 7a and Figure 7b show images of CHO cells in a culture medium with visible GoHAP90s agglomerates in the medium and internalized by the cells. We were able to record cells undergoing mitosis. Interestingly, the agglomerates internalized by GoHAP90s were distributed in both new cells. The same phenomenon was observed for other materials (images not shown). Observations confirmed that hydroxyapatite agglomerates can be easily taken up by the cells [20,23,61] (probably through macropinocytosis or phagocytosis). Figure 7c presents characteristic spherical agglomerates of hydroxyapatite F202 (concentration: 100 µg/mL) and the heavily packed cells. For comparison, Figure 7d shows control-sample cells without any hydroxyapatite addition. Confocal imaging has shown the penetration of HAp into the cells and the interesting phenomenon of enormous absorption of very large HAp nanoobjects by the cells. Statistical correlations have shown that statistically significant, with a relatively high correlation coefficient to the nanoobject absorption, are phase purity (in the context of J774.1 and BEAS-2B cell lines [p*]), crystallinity (in the context of BEAS-2B and A549 cell lines [p*]), and pH value (in the context of J774.1, A549 [p*] and BEAS-2B cell lines [p***]). Decreasing crystallinity and phase purity, as well as an increase in pH value, increase the negative effect. A very high correlation exists between the change of pH value and the viability of BEAS-2B human bronchial epithelial cells. In 90% of the cells, an increase of pH value will induce a toxic effect (based on *r*^2^). Other relationships are rather weak or average. Sizes of HAp nanoobjects correlate on a weak or average level with cell viability. *L* and *F* are on the edge of significance, but this relationship is much stronger [p*] if the correlation is applied to the distribution of NOAA sizes. A narrow distribution of a given nanoobject size may determine how easily they are absorbed by the cells. The differences in density, surface area, crystallite size, and zeta potential of the studied hydroxyapatites have no significant effect on the viability of tested cells. Particles in the group of materials with the most negative biological response had the following shapes: needle-like (F201), ellipsoidal (F202), flake-like (CaHFAP300) and spherical (HApSA+Si). When comparing all particle shapes of the examined powders, it is difficult to find a clear relationship between the shape and the cell assay results.

### Possible mechanisms of HAp NOAA–cell interactions

The diversity of physicochemical characteristics of the studied hydroxyapatites, which are the result of the different synthesis methods, allowed for an analysis of the mechanisms of NOAA interaction with the tested cells. Comparing test results with the physicochemical properties of the sample powders, a noticeable division of hydroxyapatites (in the context of a noticeable negative cellular response) into two groups can be drawn: (i) materials with elongated particles, small crystallite sizes, large surface area, high crystallinity and relatively high phase purity (F201 and F202), and (ii) materials with bigger crystallites, low surface area, low phase purity and low crystallinity (HApSA+Si, CaHFAP300). Additionally, CaHFAP300 and CaHAP300 (with high, but not toxic, negative effect on CHO and J774.1 cells) have the smallest NOAA in water, which is connected to high carbonate content, relatively high negative zeta potential and high solubility in water. It is worth to mention that when the zeta potential is higher, then the HAp–cell contact surface should be greater [62].

Confocal imaging confirmed that HAp nanoobjects were easily taken up by the cells, surprisingly enough, even those in a highly agglomerated state. This is understandable on the basis of the nature of calcium phosphates and their important functions in the organism. It is postulated that there is a much higher interaction of HAp nanoobjects with cells when they are in the interior of the cells instead of being outside in close proximity. The growth stimulation in cells is probably related to a boost of PO_4_^3−^ that are a product of HAp dissolution. Phosphate groups can be used for ATP synthesis in mitochondria [63–65]. Cell-growth inhibition could be related to the presence of a slight excess of calcium ions inside the cytoplasm, which disrupts the intracellular calcium homeostasis and inhibits cells transcriptional and translational processes [66–67], slowing down proliferation and cell growth. Calcium ions play an important role in cellular processes such as transcription, motility, exocytosis and apoptosis [68]. Intracellular calcium acts as a secondary messenger [69–70] and its concentration is strictly regulated by the cell [71]. A toxic effect occurs when more cells are dying than are produced by cell division, and is connected to apoptosis, i.e., programmed cell death. According to Ramovatar et al. [49], a high intracellular Ca^2+^ concentration may trigger a cascade of events, i.e., the activation of calpain (protein kinases), the disruption of the cytoskeletal integrity [72], the induction of stress inside the cell and the activation of the tumour-suppressor gene p53 [73–74], which promotes the downstream gene expression finally leading to an apoptosis. Gene p53 is also activated by phosphorylation, that is, the attachment of the phosphate group (PO_4_^3−^) to the protein chain [75–76].

The viability of BEAS-2B and A549 cell lines weakly depends on the presence of HAp (see Figure 6). Both cell lines maintain a high viability in the presence of hydroxyapatite even at higher concentrations (F201, F202, and HApSA+Si are exceptions at 300 μg/mL). Small concentrations of HAp in the cell environment usually stimulate cell growth. However, this is not very evident regarding the BEAS-2B viability results. Likewise, a high HAp concentration has no substantial effect on those cells, from which it can be concluded that BEAS-2B cells do not take up much of the hydroxyapatite or they remove most of the hydroxyapatite quickly. After an extrapolation of viability results for the whole range of HAp concentrations (trend line *y* = a*x* + b), the concentration value needed for a toxic effect to occur for all of the studied samples is easily determined. It turns out that relatively high doses, measured in milligrams per millilitre, are needed to cause a toxic effect (more than 50% dead cells after 24 h or complete inhibition of cell division) in human bronchial epithelial cells. F201, F202 and HApSa+Si are exceptions here, but their predicted “toxic concentrations” are still higher than in the case of other tested cell lines. This is important for the assessment of inhalation exposure connected to the processing of nanostructured calcium phosphate powders similar to the studied samples. The next stage of inhalation-safety research should be long-term tests and dustiness tests with nanoscale HAp, which were not carried out in this study.

The small impact of HAp nanoobjects on A549 cells is probably connected to their cancerous nature and lower intracellular pH value [77]. Even after a high uptake of HAp particles, A549 could dissolve hydroxyapatite more efficiently and safely than the other studied cells. CHO cells are the most sensitive to HAp particles, which is visible in the large viability differences between the samples treated low and high HAp concentrations. The effect strongly depends on the dose and the type of hydroxyapatite. For example, HAp F202 and GoHAP600s differ greatly in the presented results. Only a high concentration (300 µg/mL) of GoHAP600s has a (very small) inhibiting effect on cell growth. F202 exhibits this effect even at a small concentration (10 µg/mL), while for higher concentrations a toxic effect occurs. Surprisingly enough, their physicochemical parameters such as average surface area, size of crystals and size of agglomerates in water were very similar (see Table 2). There is a small difference in their densities (2.95 g/cm^3^ for F202 and 3.03 g/cm^3^ for GoHAP600s) and crystallinity (99.1% for F202 and 89.6% for GoHAP600s). The most vulnerable cells belong to the CHO cell line, which can be explained by the nature of the reproductive system in which calcium signalling plays a crucial role. For example, calcium signals control cell division in early embryos and are very important in the development of patterning [78]. Macrophages are cells responsible for detecting, ingesting and digesting cellular waste and microbes [79–80]. The viability results of the examined J774.1 cell line showed a small overall impact of the hydroxyapatite samples, with the exceptions of low toxicity of F202 at 300 µg/mL and a stronger toxic effect for HApSA+Si at 200–300 µg/mL. Macrophages are used to absorb foreign substances and because of their nature they have no significant difficulties digesting excess hydroxyapatite. In the case of these cells, a problem might be related to the high pH value connected to solubility of HApSA+Si and its lowest crystallinity among all of the studied hydroxyapatite powders (i.e., by disturbing calcium homeostasis). This factor depends on the stability of HAp aggregates and agglomerates, and could be also a possible reason for the high negative effect of CaHAP and CaHFAP on CHO and J774.1 cell lines.

The differences in the cell viability results may be also related to the internal pH values. As the pH value decreases, the solubility of calcium phosphates increases [81]. Paradoxically, the poor solubility and high crystallinity of F201 and F202 are the most plausible explanation of their toxicity in CHO and J774.1 cells. The narrow size distribution of their NOAA facilitates their intake by the cells. The low solubility of F201 and F202 could cause a retention of calcium phosphate deposits inside the cells, a kind of “cell stones”, similar to the deposition of macroscale calcium-phosphate stones in the kidneys. Such retention can prevent cellular processes from occurring. The solubility is also connected to the content of crystallisation water [15] of the hydroxyapatite particles. In case of F201 and F202, that crystallisation-water content may be lower in comparison to other samples because of the spray-drying treatment. Basing on same logic, NOAA of highly crystalline HApSA should also cause a similar retention effect, but F201 and F202 also had some content of very small particles the size of which is comparable to the size of DNA (ca. 10 nm). When their nanoobjects start to dissolve inside the cells, these small particles will be released in a “trojan horse” effect, where they may directly or indirectly affect transcription of genes. GoHAP90s also has a significant content of such small particles, but this hydroxyapatite has much larger NOAA size distribution, which results in a much lower concentration of objects that can be phagocytosed. In this case, the actual concentration of hydroxyapatites entering the cells is lower than overall concentration of the material added to test dishes. There are also considerable differences in SSA, crystallite sizes, density and phase purity between those nanoscale HAp materials. The “trojan horse” effect could also occur when the absorbed HAp objects contain impurities. For the evaluation of this mechanism, additional studies on degradation and particle release need to be carried out.

## Conclusion

The presented results provide useful information on nanosized hydroxyapatites obtained through different synthesis methods, their applications and short-term impact on different cells, including an attempt to explain the mechanisms behind the toxic effect. Ten nanosized hydroxyapatite samples manufactured via different methods (combustion chemical vapour condensation, a wet-chemical method with spray drying, microwave solvothermal synthesis and synthesis in molten salts conducted at 300 °C) were characterized. A range of physicochemical characteristics including particle size, shape, specific surface area, degree of crystallinity, surface charge, state of agglomeration, densities, pH value, NOAA size and stoichiometry was scrutinized for a better understanding of the interactions between nanoscale hydroxyapatite and cells. The biological impact depends on dose and physicochemical properties of the HAp particles and the cell nature. Toxic effects occur only in the case of a high overdose. The postulated mechanisms of the toxic effect are the delivery of impurities into the interior of the cell (“trojan horse” effect), the physical clogging of the cell interior (cell stones), a perturbation of calcium homeostasis by the excess of calcium ions, and the induction of apoptosis. An important finding of the present study is the considerable influence of crystallinity, solubility, phase purity and state of agglomeration of the nanoparticles on cell viability. The intermediate, but very significant, effect on the cells is due to the relationship between particle size and surface area, density, solubility and tendency to agglomeration.

By choosing appropriate synthesis methods, hydroxyapatite materials with desired physicochemical properties for specific applications can be designed. Hydroxyapatites with larger particles and lower surface area were obtained after synthesis with thermal treatment during the process. State of agglomeration and solubility are mainly influenced by the drying methods. A uniform size of agglomerates with a highly developed surface area (achieved, e.g., by spray drying) and hence better uptake by the cells can be used as an advantage for drug delivery or more effective gene transfection based on hydroxyapatite. The degree of crystallinity can be a factor in determining how long an agglomerate will stay inside the cell and what will be the drug-release rate. Hydroxyapatites with exceptionally large surface area could be also used for chromatography, protein purification, cell-culture substrates, catalyst production and waste management. Lower crystallinity, high purity and high zeta potential of hydroxyapatite nanoparticles are desired in a material intended for long-term medical use in the body. Since calcium deficiency is a feature of biological apatite along with a relatively low degree of crystallinity (and addition of magnesium, sodium, potassium, chlorine, fluorine, carbonate and few trace elements), synthesized nanoscale hydroxyapatites with such properties might find use in implants and dentistry.
